# The Influence of Process Parameters and Build Orientation on the Creep Behaviour of a Laser Powder Bed Fused Ni-based Superalloy for Aerospace Applications

**DOI:** 10.3390/ma12091390

**Published:** 2019-04-29

**Authors:** Hani Hilal, Robert Lancaster, Spencer Jeffs, John Boswell, David Stapleton, Gavin Baxter

**Affiliations:** 1 Institute of Structural Materials, Swansea University, Bay Campus, Swansea SA1 8EN, UK; R.J.Lancaster@Swansea.ac.uk (R.L.); S.P.Jeffs@Swansea.ac.uk (S.J.); 2 Rolls-Royce plc, P.O. Box 31, Derby DE24 8BJ, UK; John.Boswell@Rolls-Royce.com (J.B.); David.Stapleton2@Rolls-Royce.com (D.S.); Gavin.Baxter@Rolls-Royce.com (G.B.)

**Keywords:** nickel-based superalloy, additive layer manufacturing, laser powder bed fusion, process parameters, small punch creep testing

## Abstract

Additive Layer Manufacturing (ALM) is an innovative net shape manufacturing technology that offers the ability to produce highly intricate components not possible through traditional wrought and cast procedures. Consequently, the aerospace industry is becoming ever more attentive in exploiting such technology for the fabrication of nickel-based superalloys in an attempt to drive further advancements within the holistic gas turbine. Given this, the requirement for the mechanical characterisation of such material is rising in parallel, with limitations in the availability of material processed restricting conventional mechanical testing; particularly with the abundance of process parameters to evaluate. As such, the Small Punch Creep (SPC) test method has been deemed an effective tool to rank the elevated temperature performance of alloys processed through ALM, credited to the small volumes of material utilised in each test and the ability to sample material from discrete locations. In this research, the SPC test will be used to assess the influence of a number of key process variables on the mechanical performance of Laser Powder Bed Fused (LPBF) Ni-based superalloy CM247LC. This will also include an investigation into the influence of build orientation and post-build treatment on creep performance, whilst considering the structural integrity of the different experimental builds.

## 1. Introduction

Nickel-based superalloys offer the ability to maintain mechanical properties at higher temperatures and stresses whilst exhibiting excellent oxidation and corrosion resistance [1]. It is for these reasons that they are heavily incorporated within numerous industrial sectors, in particular power generation within the aerospace industry where they are utilised in up to 40% of components in the gas turbine engine [2].

Despite being traditionally manufactured through cast and wrought methodologies, the aerospace industry is investing significant effort in the use of powder processing technologies for both novel component design and repair, allowing scope for innovation in terms of engine architecture and the ability to push engines to higher stresses and temperatures, thus improving the efficiency of the engine [3]. Additive Layer Manufacturing (ALM) and specifically Laser Powder Bed Fusion (LPBF) are of interest since these technologies allow scope for more complex alloy design alongside the ability to fabricate highly intricate components [4]. Given the relatively new nature of additive manufactured components, a detailed understanding of key process variables and their influence on microstructure and mechanical performance is still yet to be fully understood for exploitation in critical applications [5].

**Figure 1:**
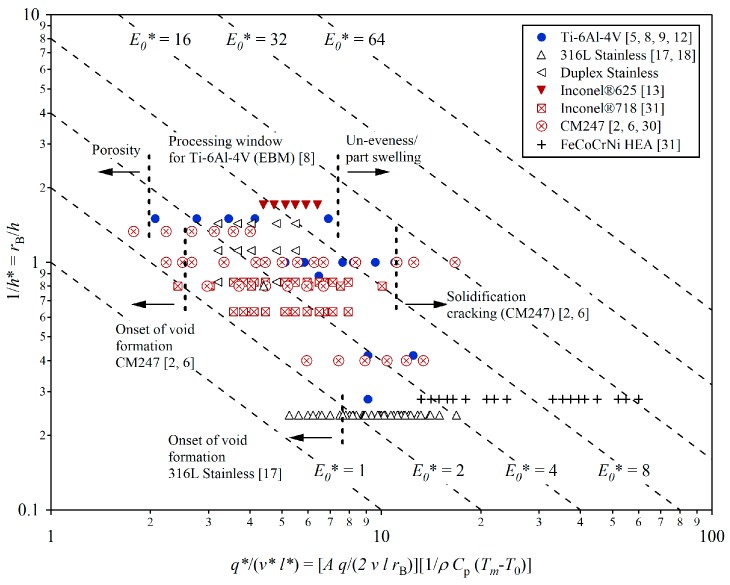
Normalised processing diagram for a range of Additive Layer Manufacturing (ALM) alloy systems, reproduced from Reference [6].

Thomas et al. [6] developed the normalised process diagram as a means of illustrating the process window for various ALM alloying systems including Ti, Ni and steel alloys, where the vertical axis acts as a function of normalised hatch spacing and the horizontal axis as the remaining key process variables (beam power, beam velocity, and layer height), as reproduced here in Figure 1 with their relevant notations indicated in Table 1. Diagonal isopleths indicate the varying degrees of energy density (E*), based on these key process variables, with the top right region being considered high energy and the bottom left considered low energy. Carter et al. [7] established that for high γ′ Ni-based alloys fabricated through LPBF, the process window is extremely narrow. High energy densities have given rise to multiple microcrack forming mechanisms, namely solidification cracking and ductility dip cracking. Conversely, low energy densities have been shown to promote microvoid formation as a result of the energy density not being sufficient enough to reach the minimum energy threshold and subsequently achieve full powder melting and consolidation. 

This narrow process window is further complicated due to the highly sophisticated microstructures which are typically produced through additive technologies, where the layer-by-layer melting and re-melting of subsequent layers of powder, promotes heavily textured, epitaxial grain growth leading to anisotropic microstructures [8]. Many authors have detailed the difficulties in establishing appropriate test methodologies for a mechanical assessment of such materials due to the highly transient microstructures, build discontinuity features, and the inherent lack of consistency in the desired final component [8,9], thus potentially making traditional uniaxial testing approaches inefficient. To overcome such issues, recent mechanical test approaches have typically required either building a separate test specimen within the build volume or alternatively extracting a test piece from within a fully-built component. These methods, however, are inadequate for characterising the local microstructures of small complex features due to the geometrical dependency on mechanical properties and the volume constraints of the features which are of most interest. Therefore, the use of small scale testing techniques offers the unique potential of highly localised mechanical property assessment. One of the most established small scale test methodologies is the Small Punch (SP) test, a mechanical test approach that can be employed to characterise the creep [10,11], tensile [12], fracture [13], and more recently, fatigue properties [14] of metallic materials. In more recent years, SP testing has found increasing use in the aerospace industry, particularly for characterising novel experimental materials where material quantities are restricted. In addition, significant effort has been made in realising the benefits of SP testing for assessing the properties of ALM materials, including LPBF Ni-based superalloy C263 [15] and Electron Beam Melted (EBM) Ti-6Al-4V [16].

In this research, the SP creep test will be employed to mechanically assess and rank the creep behaviour of LPBF variants of the Ni-based superalloy, CM247LC. Previous studies have analysed the creep properties of directionally solidified CM247LC [17,18], however there is no SPC or conventional creep data currently available for an additively manufactured derivative of the alloy in these build orientations. Studies will focus on the influence of the key process variables, build orientation, and post-manufacture treatment on high-temperature properties, alongside detailed microstructural characterisation, to reveal the most prevalent factors on creep performance. 

## 2. Materials and Methods

### 2.1. Material 

**Figure 2:**
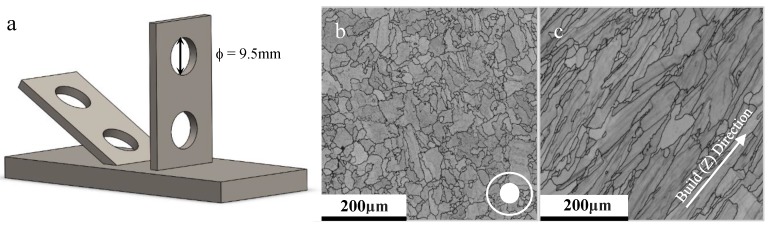
(**a**) 30° and 90° build orientations with sectioning locations, (**b**) 30° build microstructure, and (**c**) 90° build microstructure.

**Figure 3:**
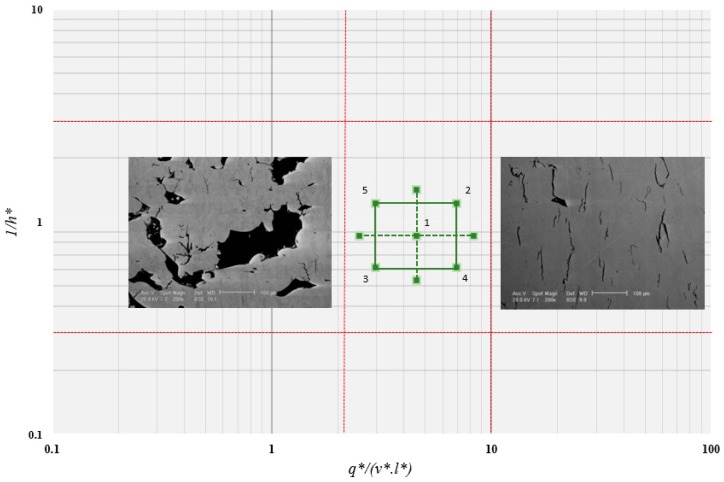
Normalised process parameter map with sample parameter set identification and indication of expected microstructural features outside the process window [6,7].

The high-temperature properties of a series of LPBF CM247LC variants were the focus of this study. A nominal composition of the alloy is presented in Table 2. The LPBF materials were built as flat plates in an EOS M280 machine (EOS, Munich, Germany) from ATI’s gas atomised powder, with base process parameters broadly in line with industry standards. The plates were built in one of two primary orientations, 30° or 90°, with illustrative microstructures in the Y-Z plane for each given in Figure 2b,c. The plates were built under a variety of parameter sets in accordance with the process window as depicted in Figure 3 and Table 3. Here, q* is normalised beam power, v* is normalised beam velocity, l* is normalised layer height, h* is normalised hatch spacing and E* is energy density (J/mm^2^). Further detail on the derivation of these parameters is given in Reference [6].

In addition to build orientation and the process parameters, post-processing conditions have been shown to have a major influence on microstructure and the subsequent mechanical performance [8]. Heat treatments (HT) can be introduced to manipulate the microstructure for the benefit of the component’s desired properties. Here, given the material’s utilisation in high-temperature applications, a coarser grain morphology with a fine dispersion of γ′ precipitates is preferred to aid creep resistance, as such, heat treatments were performed above the γ′ solvus temperature to alter the shape, size, and distribution of the precipitates. Similarly, hot isostatic pressing (HIP) has been shown to effectively ‘heal’ internal cracks, removing possible initiation sites for associated failure mechanisms [19]. Therefore, these additional post-processing conditions were compared against the as-built equivalent. In this case, the material is subjected to a bespoke HIP cycle at 1220 °C, 143 MPa for 2 h below the γ′ solvus. From here, a solution HT at 1250 °C is conducted for 4 h to allow sufficient time for γ′ to form a solid solution. This is subsequently followed by an aging treatment at 870 °C for 16 h in order to prevent changes to the carbide morphology and distribution.

A series of six plates for each parameter set was fabricated using ATI’s CM247LC powder (powder particle size distribution [d50 ≈ 30 µm, Dv50 ≈ 1.52 µm]), with two 9.5 mm diameter cylindrical samples extracted from each, via Electron Discharge Machining (EDM), as represented in Figure 2a. Of the two specimens, one was left in the as-built (AB) condition and the other subjected to a series of alternating heat treatments and hot isostatic pressing (HT/HIP) conditions as previously discussed. The φ9.5 mm rods were then progressively ground and polished down to an SP disc with a thickness of 500 µm +/−5 µm with 1200 grit finish, as dictated by the Small Punch European Code of Practice (CoP) [20]. The LPBF variants were then run through standard metallographic preparation procedures for nickel-based superalloys and characterized using scanning electron microscopy (SEM) and electron back-scatter diffraction (EBSD). For SEM analysis, samples were etched for 10 seconds using Kallings 2 reagent following a four-stage polishing procedure. For EBSD analysis, a step size of 0.25 µm was used alongside HKL Channel 5′s elliptical fit method.

### 2.2. Small Punch Creep Testing

The SP creep tests were performed on an elevated temperature SP creep frame developed at Swansea University, in accordance with the CoP [20,21]. In this test arrangement, the miniature disc specimen is fixed in location between an upper and lower die, securely clamping the specimen in place. Loading is applied through the central axis of the test frame, via an upper load pan arrangement with weighted loads, in line with a 2 mm diameter hemispherical ended alumina ceramic punch. Heat was applied using a single zone digitally controlled furnace and was maintained to within ±1 °C of the test temperature of 950 °C. Temperature was constantly monitored throughout the test by two Type N thermocouples located in a drilled hole in the upper die assembly, close to the surface of the disc. All tests were performed in an inert argon gas environment that was encased with a ceramic tube, as seen in Reference [21], in order to eliminate any potential oxidation effects that may reduce creep life. The utilisation of cooling jackets and PTFE seals were incorporated to further prevent any possibilities or argon leakage. Two linear variable displacement transducers (LVDTs) were utilised on either side of the disc to monitor the deformation; one transducer located directly below the load pan to measure the displacement upon the top surface of the disc, the other transducer measuring displacement directly from the base of the disc via a quartz rod. Deformation was recorded using designated in-house logging software. All tests were performed under a fixed load of 150 N.

## 3. Results and Discussion

**Figure 4:**
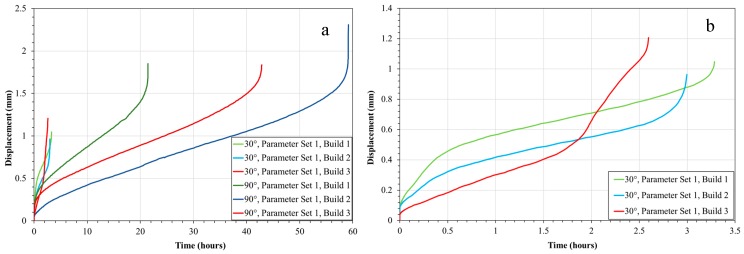
(**a**) Small Punch (SP) creep tests of 30° vs. 90° build orientations; (**b**) 30° build orientation results.

SP creep results were gathered across all build variables with Figure 4 presenting a direct comparison between build orientations for the same parameter set in the post-processed condition, where the 90° orientation offers a significantly superior creep performance to the 30° equivalent. Since creep deformation is a grain boundary initiated and dominated mechanism, this can be directly related to the differing grain structure, with larger elongated grains more prominent in the 90° build due to epitaxial grain growth mechanisms, and as such a lower volume fraction of grain boundaries, whereas the 30° orientation appears more akin to an equiaxed morphology consisting of smaller grains. This is supported by the grain size measurements presented in Table 4, where the aspect ratios across the two orientations also differ.

**Figure 5:**
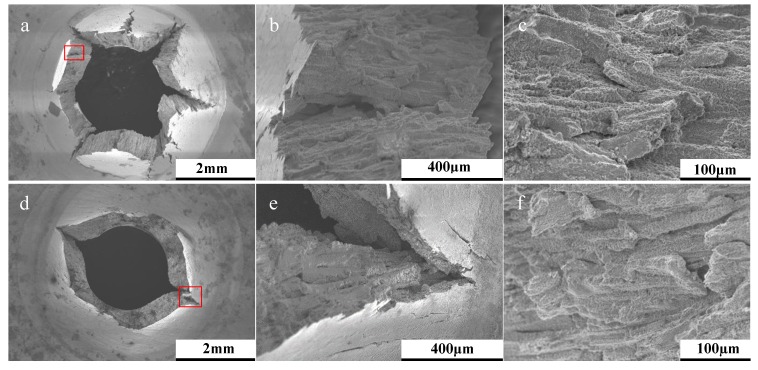
Post-rupture fractography of (**a**–**c**) 30° build orientation and (**d**–**f**) 90° build orientation.

Post-rupture fractography revealed the contrasting failure modes across the two orientations, with examples depicted in Figure 5. The image presents the failure behavior for two build 3 specimens, showing that the 90° disc experienced a form of fracture directly controlled by the underlying microstructure, as evidenced by the directionality of the failure, which is aligned parallel to the epitaxial columnar grain structure. The 30° equivalent (Figure 5a–c) offers a more brittle type response with ‘starfish’ radial type cracking emanating from the centre of the disc, thus leading to a reduction in the material’s resistance to creep. This is related to the microstructure of the 30° builds, where the grain morphology of the punch contact surface is predominantly equiaxed (Figure 1b), and given that the SP disc geometry is only 500 µm in thickness, the influence of epitaxial grain growth is restricted. Therefore, microstructural investigations were furthered for the 90° builds. A common feature seen across the failures of the two build orientations is the level of intergranular damage around the columnar grains, suggesting similar failure mechanisms are taking place. However, for this example, the orientation of the grains is seen to dictate the overall creep behaviour of the specimens.

**Figure 6:**
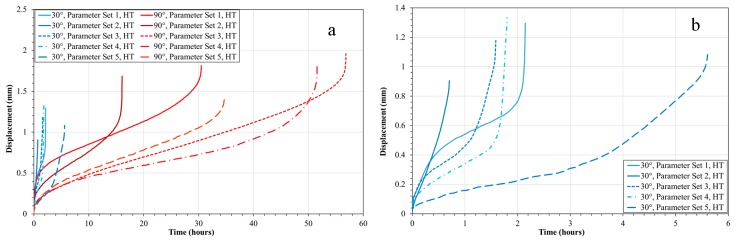
(**a**) SP creep results for 30° and 90° builds across the five Parameter Sets; (**b**) 30° build orientation results.

**Figure 7:**
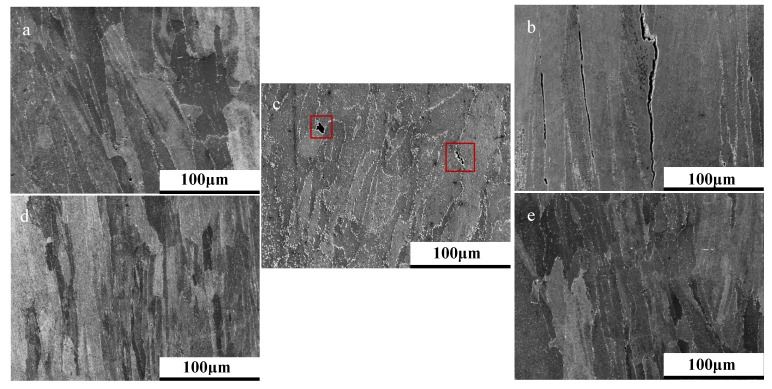
Microstructural features of 90° specimens for the 5 different parameter sets (**a**) 5, (**b**) 2, (**c**) 1, (**d**) 3, (**e**) 4 in relation to the DOE testing matrix given in Figure 3.

In addition to build orientation, process variables and subsequently energy density are also seen to have an impact on SP creep performance. As can be seen in Figure 6, when comparing alternative parameter sets across the two build orientations, all of which were subjected to the post-build HIP/HT, the 90°, Parameter Set 3 (low E*) condition provides the greatest creep resistance with a time to rupture of approximately 57 h. Conversely, the 90° Parameter Set 2 material (high E*) displays the worst time to rupture, failing after approximately 16 h, with Parameter Set 1 (medium E*) giving an intermediate response and failing after 21.5 h. The root cause of the variation across the parameter sets is the subtle differences in the microstructure. Figure 7 displays the microstructures for the 5 different parameter sets, built in the 90° orientation, with the microstructure in each representing the plane in which the load is exerted during SP creep testing. Despite the noticeable influence of process variables on grain sizing and coarsening as previously discussed, the process parameters appear to influence the formation and presence of material discontinuities. The 90°, Parameter Set 3 (low E*) HT displays very little or infrequent features in the microstructure. However, as energy increases, early signs of grain boundary cavitation can be seen, as shown in Parameter Set 1 (Figure 7c.), which can lead to an appreciative decrease in high-temperature mechanical performance. Furthermore, as energy density reaches the high end of the testing matrix, cavitation coalescence seems to occur, resulting in an intergranular fracture as shown, consequently further reducing creep performance.

A further key process variable to consider in Figure 7 is the influence of hatch spacing. Upon comparing 90°, Parameter Sets 4, (Figure 7e. low 1/h*) and 5 (Figure 7a. high 1/h*), time to rupture varies from 52 h to 35 h, respectively. Previous literature has suggested that high 1/h* values on an ordinate scale would lead to track re-melting and subsequently give rise to grain coarsening effects whilst low 1/h* could lead to a lack of re-melting and give rise to poor material consolidation [22]. In this instance, neither of these microstructural phenomena are witnessed which could be due to numerous factors. One explanation is the nature of SP creep testing regarding small specimen geometries, since the test arrangement gives rise to elastic heterogeneity as a result of extremely localised testing. In this instance, given the small 9.5 mm diameter specimens, a localised area of grain structure could have been suspected to testing, and therefore may not give an accurate representation as to the bulk properties of such parameter sets. Given the relatively consistent nature of results across the 30° build orientations, from a microstructural standpoint, there tends not to be much variation.

**Figure 8:**
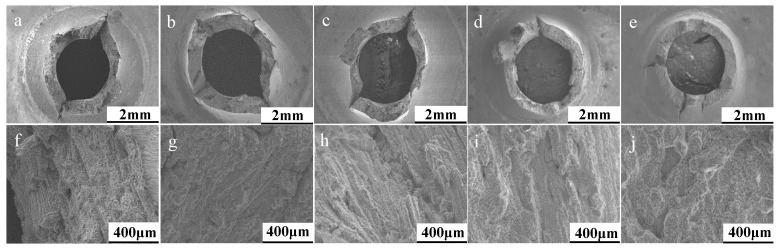
SP creep fractography across DOE parameter sets for 90° build orientations. Parameter set (**a**,**f**) 1, (**b**,**g**) 2, (**c**,**h**) 3, (**d**,**i**) 4, (**e**,**j**) 5.

Fractographic analysis in Figure 8 shows that across the different parameter sets for the 90° specimens, the variation in levels of embrittlement is somewhat negligible given the lack of ‘starfish’ type cracking previously seen, which in combination to the faceted fracture surfaces observed at higher magnifications, suggests that consequently, there is not a difference in the failure mechanism, rather a rate of failure.

**Figure 9:**
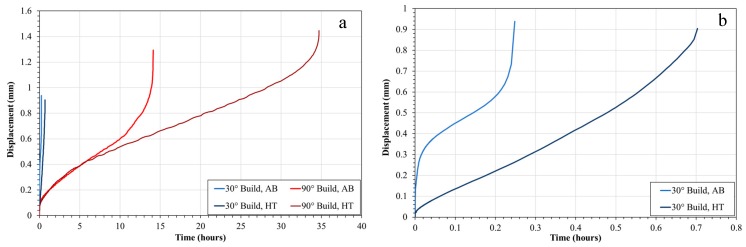
(**a**) SP creep of 30° and 90° samples both in the as-built and post-processed condition; (**b**) 30° build orientation results.

**Figure 10:**
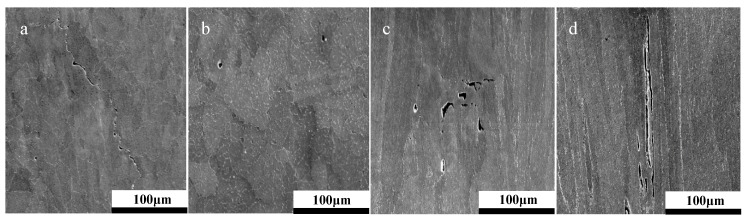
Microstructure of 30° Parameter Set 2 (**a**) as built (AB) and (**b**) heat treated (HT), and 90° Parameter Set 2 (**c**) AB and (**d**) HT.

In addition to process variables such as build orientation and parameter selection, post-processing treatments are shown to be a pivotal means for microstructural manipulation and subsequently creep performance, as shown in Figure 9. Regardless of build orientation, alternating post-processing procedures such as solution heat treatments and hot isostatic pressing enhance resistance to high-temperature mechanical deformation. This can be explained as a result of the microstructural changes observed in Figure 10, where signs of cavitation coalescence and frequent hot tearing can be witnessed in the as-built state. In the HIPed and heat treated condition, it can be seen that this coalescence is less prevalent at 30° and despite intergranular cracking observed at 90°, they are far less prominent than the hot tearing previously seen.

**Figure 11:**
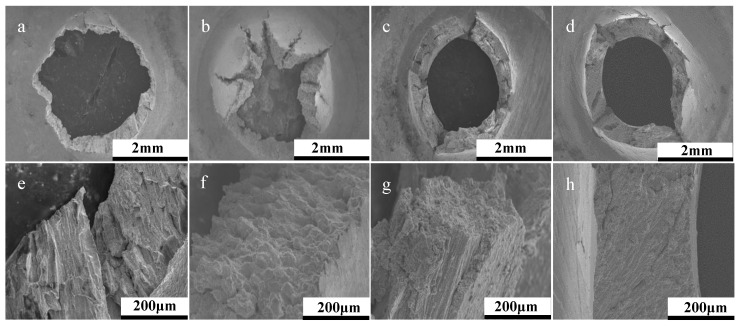
Post-rupture fractography of 30° Parameter Set 2 AB (**a**,**e**), HT (**b**,**f**), and 90° Parameter Set 2 AB (**c**,**g**) and HT (**d**,**h**).

**Figure 12:**
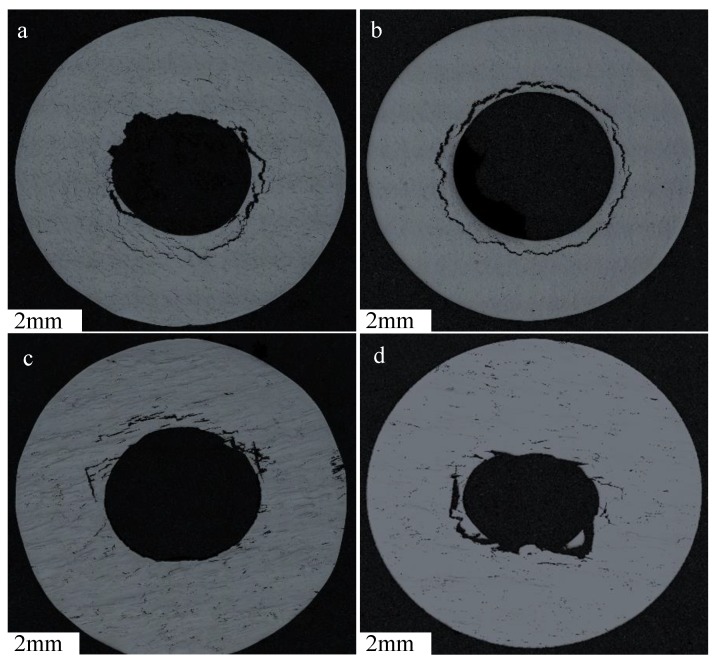
Circumferentially clamped regions of 30° and 90° Parameter Set 2 AB (**a**,**c**) and 30° and 90° Parameter Set 2 HT (**b**,**d**).

Figure 11 illustrates that post-processing treatments do not appear to influence the fracture mechanisms associated with these failures. Figure 12 and Table 5 can further validate the influence of post-processing conditions on the presence of material discontinuities, with the volume fraction of porosity and intergranular cavitation reducing with the introduction of post-process treatments. Porosity and microcracking information were calculated using ImageJ thresholding, where the determinative criteria for the difference between a crack and a pore were set at a circularity value of 0.5.

**Figure 13:**
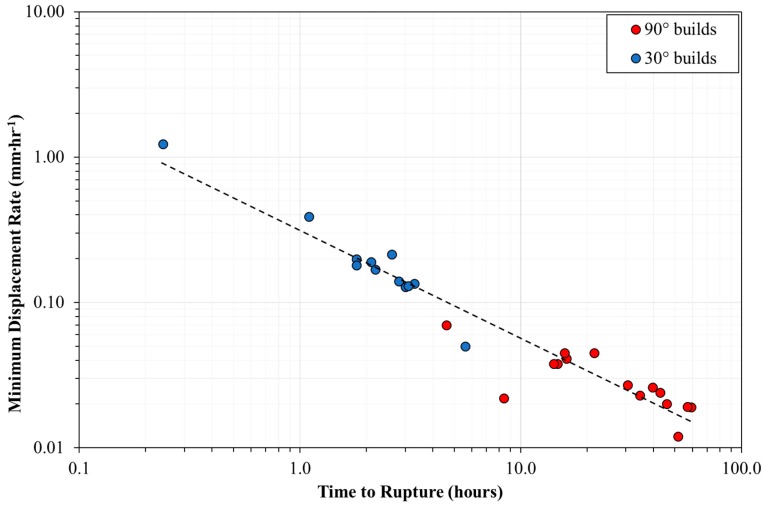
The modified Monkman-Grant relationship for all LPBF CM247LC samples tested at 950 °C, 150 N load.

Despite the factors discussed playing a role on the elevated temperature creep properties of LPBF CM247LC, a final consideration should be made regarding the consistency of the test. Previous literature has demonstrated the validity of the Monkman-Grant relationship for small punch test data [23]. Figure 13 displays a modified Monkman-Grant relationship for the suite of tests conducted within this research, with a clear linear relationship and correlation between Time to Rupture and Minimum Displacement Rate. Limited scatter is apparent, supporting the repeatability of the test arrangement, whilst it is also evident that 30° builds exhibit a far inferior resistance to high-temperature mechanical deformation.

## 4. Conclusions

In this research, a series of LPBF CM247LC samples of varying build orientation, parameter sets, and post-processing conditions were mechanically assessed through small punch creep testing. Build orientation has been shown to be the primary influence on the material’s creep resistance at high temperature, exhibiting strong anisotropic behavior due to epitaxial grain growth in the build direction. The 30° builds display a fine equiaxed grain structure and subsequently display a poor creep performance given the high-volume fraction of grain boundaries, whereas the 90° builds offer a coarsened columnar grain structure and thus performed significantly greater under creep deformation.

In addition to build orientation, the impact of energy density, a value based upon key process parameters (beam power, beam velocity, layer height and hatch spacing), on high-temperature mechanical performance was investigated. It has been shown that lower energy densities that surpass a minimum threshold are preferable, given that little to no material discontinuities were present. However, as energy density increases, early signs of cavitation are observed, which will eventually coalesce as they reach the high end of the spectrum, giving rise to intergranular fracture. Furthermore, variations in hatch spacing at a consistent energy density were also investigated, with low hatch spacing expectedly performing better, despite the re-melting and coarsening effect. This is attributed to elastic heterogeneity that can be witnessed in localised testing methodologies such as small punch creep testing, where differing grain structures that are not representative of bulk properties are observed.

Finally, alternating post-processing treatments such as solution heat treating, and hot isostatic pressing were compared and contrasted with samples in the as-built condition. It was found that regardless of the build orientation, post-processing treatments were an effective tool in the enhancement of high-temperature mechanical performance as a result of microstructural improvement through the healing of material discontinuities.

## Figures and Tables

**Table 1:** Process and material variables that contribute to process parameters.

Notation	Process Variables & Material Properties	Units	Notation	Process Variables & Material Properties	Units
*A*	Surface Absorptivity	-	q*	Normalised Power	-
*C_p_*	Specific Heat Capacity	J kg^−1^ K^−1^	*rB*	Beam Radius	M
E_0_*	Normalised Energy Density	-	*T_m_*	Melting Temperature	K
*h*	Hatch Spacing	m	*T_0_*	Initial Powder Bed Temperature	K
h*	Normalised Hatch Spacing	-	*v*	Beam Velocity	ms^−1^
*ρ*	Density	kg m^−3^	V*	Normalised Beam Velocity	-
*q*	Power	W			

**Table 2:** Typical chemical composition of CM247LC (wt%).

**C**	**Cr**	**Ni**	**Co**	**Mo**	**W**	**Ta**
0.07	8	Bal.	9	0.5	10	3.2
**Ti**	**Al**	**B**	**Zr**	**Hf**	**Si**	**S**
0.7	5.6	0.015	0.01	1.4	0.03	15 ppm

**Table 3:** Design of experiments (DOE) parameter set testing matrix.

DOE Parameter Set	q*/(v*·l*)	1/h*	E*
1	Medium	Medium	Medium
2	High	High	High
3	Low	Low	Low
4	High	Low	Medium
5	Low	High	Medium

**Table 4:** Grain size measurements at 250x magnification taken using electron back-scatter diffraction.

Sample ID	Average Grain Area (μm^2^)	Average Grain Aspect Ratio	Average Grain Diameter (µm)	Number of Grains Analysed
**30°, Parameter Set 1, HT**	175	1.90	9.9	1334
**90°, Parameter Set 1, HT**	863	3.79	20.4	378
**90°, Parameter Set 2, HT**	811	3.67	13.0	477
**90°, Parameter Set 2, AB**	1278	3.69	21.5	284
**90°, Parameter Set 3, HT**	1024	2.98	19.7	311
**90°, Parameter Set 4, HT**	964	3.43	20.1	337
**90°, Parameter Set 5, HT**	1129	2.74	21.4	299

**Table 5:** Microcracking and porosity calculations for post-processing conditions.

Sample	Microcracking (% Area)	Porosity (% Area)
**30°, Parameter Set 2, AB**	1.70	0.51
**30°, Parameter Set 2, HT**	0.31	0.50
**90°, Parameter Set 2, AB**	1.00	0.35
**90°, Parameter Set 2, HT**	1.67	0.33
